# Social Mechanisms to Get People Outdoors: Bimodal Distribution of Interest in Nature?

**DOI:** 10.3389/fpubh.2016.00257

**Published:** 2016-11-15

**Authors:** Ralf Christopher Buckley, Diane Westaway, Paula Brough

**Affiliations:** ^1^Griffith University, Gold Coast, QLD, Australia; ^2^Coastrek, Sydney, NSW, Australia; ^3^Menzies Health Institute of Queensland, Griffith University, Gold Coast, QLD, Australia

**Keywords:** policy making, psychology, social, outdoors, exercise therapy, nature relatedness

## Abstract

We report results from a post-program survey (*n* = 930) of participants in a non-profit outdoor health program targeted principally at women with families in Australia’s metropolitan cities. We analyze communications, motivations, experiences, satisfaction, and intentions. The program involves 3 months’ outdoor training in scenic locations, culminating in a single-day event. Training includes social opportunities and peer-group support. Event entry is in teams and includes charitable fundraising and personal challenges. Drop-out rates are very low, and repeat sign-up high. There are 2,000–3,600 places per event, and the most recent sold out in <24 h. We propose that for urban residents of developed nations, individual interest in exposure to nature may be bimodal rather than unimodal. Programs of this type target individuals most likely to shift from low-interest to high-interest mode, using a set of social levers to change attitudes and behaviors. This contrasts with most public outdoor health programs, which assume a unimodal distribution and aim for small lifestyle changes at population scale. We suggest that the bimodal hypothesis is relevant to the sociocultural context of psychosocial interventions in a public health context, and merits direct testing.

## Introduction

For urban residents of wealthy temperate developed nations, who normally work indoors, outdoor exercise in unpolluted natural areas improves both physical health (1–7) and mental health (8–16). Mental health gains from exposure to nature include reduced stress, anxiety, and depression (17, 18); and improved sleep (19, 20) and cognition (21–23). These mental health improvements are non-specific: evidence indicates gains across all age groups (24–28), for both women and men (29); and across countries and cultures (27, 30, 31).

From the perspective of public health agencies and health insurers, nature provides free prevention and treatment for many health conditions: free, because other government agencies manage parks and greenspace, and individuals bear the financial costs of accessing them. Therefore, health agencies and insurers in many countries have repeatedly adopted programs intended to get people outdoors, including educational campaigns, publicly funded outdoor exercise programs, and so-called green prescription systems linked to health insurance and clinical medicine systems (32–34). Local governments also include and fund outdoor activities in their urban parks, and national parks agencies promote programs such as “Healthy Parks, Healthy People” (35) and “Every Kid in a Park” (36). Such publicly run programs have been broadly positive, but both uptake and outcomes have been rather limited in scope and duration.

Here, we present an evaluation of an alternative approach run by a non-profit organization and suggest possible reasons for its outcomes to date. The evaluation is based on post-program questionnaires completed by participants, so it relies on their perceptions and recollections, rather than paired measurements prior and subsequent to participation. As with many such studies, however, it is these perceptions and recollections that influence subsequent participant attitudes and behaviors, including repeat enrollments, so this design is appropriate for the question addressed. The possible reasons for success include the broad hypothesis that individual interest in nature may be bimodal rather than unimodal. We put forward this hypothesis for rigorous testing in future.

## Materials and Methods

### Program, Site, and Scale

The program evaluated here is known as Coastrek. It is a privately operated portfolio of annual walking events and prior training programs, held along public hiking trails around major metropolitan centers in Australia. The annual events are 30–60 km in length. The formal training programs are conducted over 3 months prior to each event. Marketing approaches are closely linked into a multi-tier network of small-scale health and fitness providers in each region. The overall approach combines multiple social measures that (a) target individuals likely to change their lifestyles to increase their exposure to nature and (b) encourage them to make this change.

The Sydney Coastrek started in 2009 with 800 individual participants, increased to 3,600 in 2016. The Melbourne Coastrek began in 2015 with 1,300 participants and increased to 2,400 in 2016. The Sunshine Coastrek will start in 2017 with 2,000 places. Overall, 90% of participants in the Coastrek program are females. Participants sign up 6 months in advance of the walk, and places are taken rapidly. For Melbourne in 2016, for example, online registration to the entire event sold out to previous Coastrek participants within 24 h, so the organizers had to negotiate additional places to provide for new participants.

### Data and Analysis

We distributed an online post-event survey to all 3,600 participants in the 2016 Sydney Coastrek. The survey contained 20 multiple-choice questions developed by the authors, assessing the key factors associated with the event, namely communications, motivations, experiences, satisfaction, and intentions. Responses were in categorical or rating-scale (Likert-type) format, and the Likert-type questions contained five response categories. We received *N* = 930 completed responses, a response rate of 26%. Not all respondents answered all the questions, but overall there was a minimum per-question response rate of 87%. We analyzed this dataset using straightforward parametric statistics, to identify overall response patterns, and significant associations between the various components of the questionnaire.

## Results

### Communications, Motivations, Satisfaction, and Intentions

In total, 69% of respondents (*n* = 642) reported that they had initially heard of the Coastrek program from friends and family members, i.e., *via* direct word of mouth. Repeat Coastrek participants comprised 15% (*n* = 140) of the total sample. The two key motivations for enrolling in the Coastrek program were taking part with friends (45%, *n* = 419) and the personal challenge of completing the event (41%; *n* = 381). Over 95% of respondents (*n* = 884) reported their overall experience in either the highest (“awesome”) or second-highest (“good”) Likert categories, with <1% of respondents scoring jointly in the two below-average categories. The event received high commendations (85 to 95% rated “good” or “very good”) for organization, registration, atmosphere, support, facilities, and checkpoints, respectively; and 77% (*n* = 716) of participants indicated they planned to participate again the following year. Eight different communication channels were used to maintain contact with participants, including various email formats, websites, social media, phone assistance, and briefings in person. All of these communication channels received high commendations.

### Training and Prior Participation

Across all respondents, 40% (*n* = 372) participated in the 12-week pre-event training programs, either through online instructions or through associated trek guiding companies in Sydney. A similar proportion (39%, *n* = 363) reported that they had no prior experience in any similar event. The proportion of participants taking advantage of Coastrek-sponsored training options was 49% (*n* = 456) for those who had no prior experience in similar events, and 38% (*n* = 353) for those who did have prior experience. This association is significant at *p* = < 0.001 (Fisher’s Exact Test, *n* = 567). That is, prior experience was a statistically significant but relatively weak determining factor in whether or not participants used training programs offered or endorsed by Coastrek. The remaining 60% of Coastrek participants carried out any training independently. A similar proportion (61%) reported that they had previously participated in at least 1 of 10 broadly similar walks, runs, or similar events. However, less than 1% (*n* = 9) of respondents indicated they were competitive trail-runners. That is, runners do not consider Coastrek to be a race event.

### Marketing

Marketing is targeted particularly at women of moderate fitness with limited time, including women with families. The Coastrek program is run by an Australian-based womens’ fitness and adventure company, whose marketing tagline is that it “inspires women to transform their lives” (37). The training program and the event are marketed as enjoyable self-paced social occasions in scenic locations, with ample support, sharing, and opportunities for refreshment. The relevant text on the home page (37) states: “When you sign up for Coastrek, you embark on months of adventure – planning, preparation, training, fundraising, chatting, walking, shopping….” The event is run as a challenge, not a race. It raises funds for a well-regarded and non-political international charity, but fundraising is non-competitive. Entry is in teams of four, with at least two women per team; and with social support for training, group cohesion through branded clothing, and peer pressure against dropping out.

This combination of factors creates multiple encouragements and mechanisms to sign up; multiple social incentives to continue; multiple disincentives to drop out; and multiple individual rewards, including improved physical and mental health, social opportunities and social capital, altruistic “warmglow” factor, and enhanced self-esteem. A single enthusiast in each team can convert three other individuals from a negative or neutral attitude to nature exposure, to a positive attitude and behavior. The event home page (37) states: “Your energy and enthusiasm will radiate from you as you are motivated and inspired by your challenge.”

## Discussion

**Figure 1:**
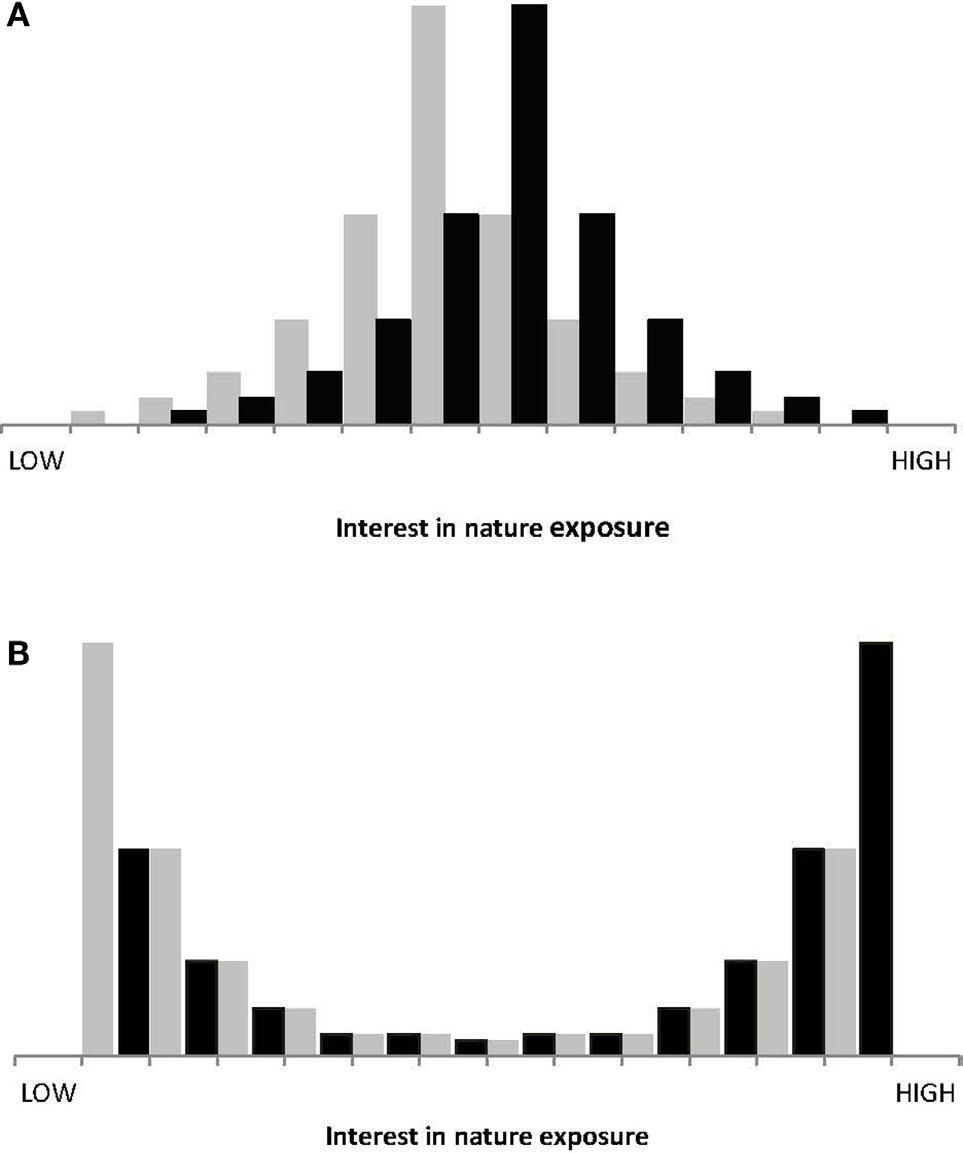
**Conceptual distributions of individual in nature exposure**. Gray: prior to public health intervention. Black: after intervention. **(A)** Current unimodal view. **(B)** Alternative bimodal view. All bar heights are conceptual only.

Past public policy measures, intended to increase individual exposure to nature, have implicitly assumed that the distribution of individual interest in nature-based outdoor activities across the population concerned is unimodal (Figure 1). They assume that population-scale education, encouragement, or incentives will lead everyone to increase exposure to nature by a small marginal amount, with a large aggregate net effect. These get-into-nature programs thus have the same underlying rationale as successful public health initiatives such as infant vaccinations and fluoridation of drinking water.

It is possible, however, that in fact the population-scale distribution of individual interest in nature may be highly bimodal, with some individuals heavily addicted to nature-based outdoor activities, and others indifferent or indeed repelled by them (Figure 1). This hypothesis has apparently not been advanced previously, and has not yet been tested, either by this study or independently.

If the bimodal hypothesis proves correct, then policy initiatives would perhaps prove more successful if they focused on moving individuals from the negative to the positive mode: a large change in attitude for a small proportion of the population, rather than a small change for a large proportion. This approach would require first, identifying and targeting specific individuals most likely to make that move; and second, identifying and applying the most effective and cost-efficient social levers to induce them to actually do so. That is a very different policy design from those adopted historically and indeed currently.

In line with this hypothesis, we suggest that one possible reason for Coastrek’s success to date may be that all of its components operate jointly to move individuals from a negative to a positive mode of attitude to nature exposure. Most Coastrek participants are busy urban women with families, who allocate little time to outdoor activities. The Coastrek program provides them with incentives, personal rewards, peer support, and social justification to include outdoor activities in nature as part of their regular schedule, displacing a part of their previously higher priorities.

None of the individual marketing approaches is new, but the combination appears to be especially effective. The 3-month preparation period creates sustained behavioral change, with nature-based adventures becoming part of participants’ regular lifestyles. For many participants, this change appears to be multi-year in length, as shown both by stated intentions, and by the 100% take-up of the 2,000 initial places in the 2016 Melbourne Coastrek, by previous Coastrek participants.

## Conclusion

From a theoretical perspective, the success of Coastrek lends at least preliminary support to our hypothesis that the population-scale distribution of interest in nature exposure may be bimodal rather than unimodal. It would now be valuable to test this hypothesis directly. This would require construction, validation, and trialing of an attitudinal and stated-behavior scale related specifically to nature-based outdoor activities; and application of this scale across large-scale random population samples, together with standard socioeconomic and demographic parameters, individual history of outdoor activities, information on outdoor activities by family, close friends, and colleagues; and data on outdoor nature-based opportunities nearby.

From a practical public health perspective, the popularity of the Coastrek model among its participants, and its ability to change their lifestyles to a more active outdoor mode, indicate the value of expanding and emulating elsewhere. In particular, its focus on adult women of moderate fitness, and particularly those women with children, enhances its public health outcomes by creating changes in diet and activity schedules for their entire families. We suggest that Coastrek provides a model that can be scaled up, expanded internationally, extended into different outdoor activities, and adopted broadly in public health policy.

## Author Contributions

RB conducted analyses and is the principal author. DW provided access to participants for data collection and assisted in interpretation. PB provided literature and expertise and contributed to writing.

## Conflict of Interest Statement

DW is the founder of Coastrek. The other authors declare no conflict of interest.
